# Supplementary material for: The interactome of intact mitochondria by cross-linking mass spectrometry provides evidence for coexisting respiratory supercomplexes
Source: Mol Cell Proteomics. 2017 Dec 8;17(2):216–32. doi: 10.1074/mcp.RA117.000470 (PMC5795388; doi:10.1074/mcp.RA117.000470)
Supplement: Supplemental Data [file supp_RA117.000470_133922_0_supp_23978_fzffwf.zip › spectra_annotation/mito_DR_spectra_annotation/310-1-10-1-6-1.pdf]

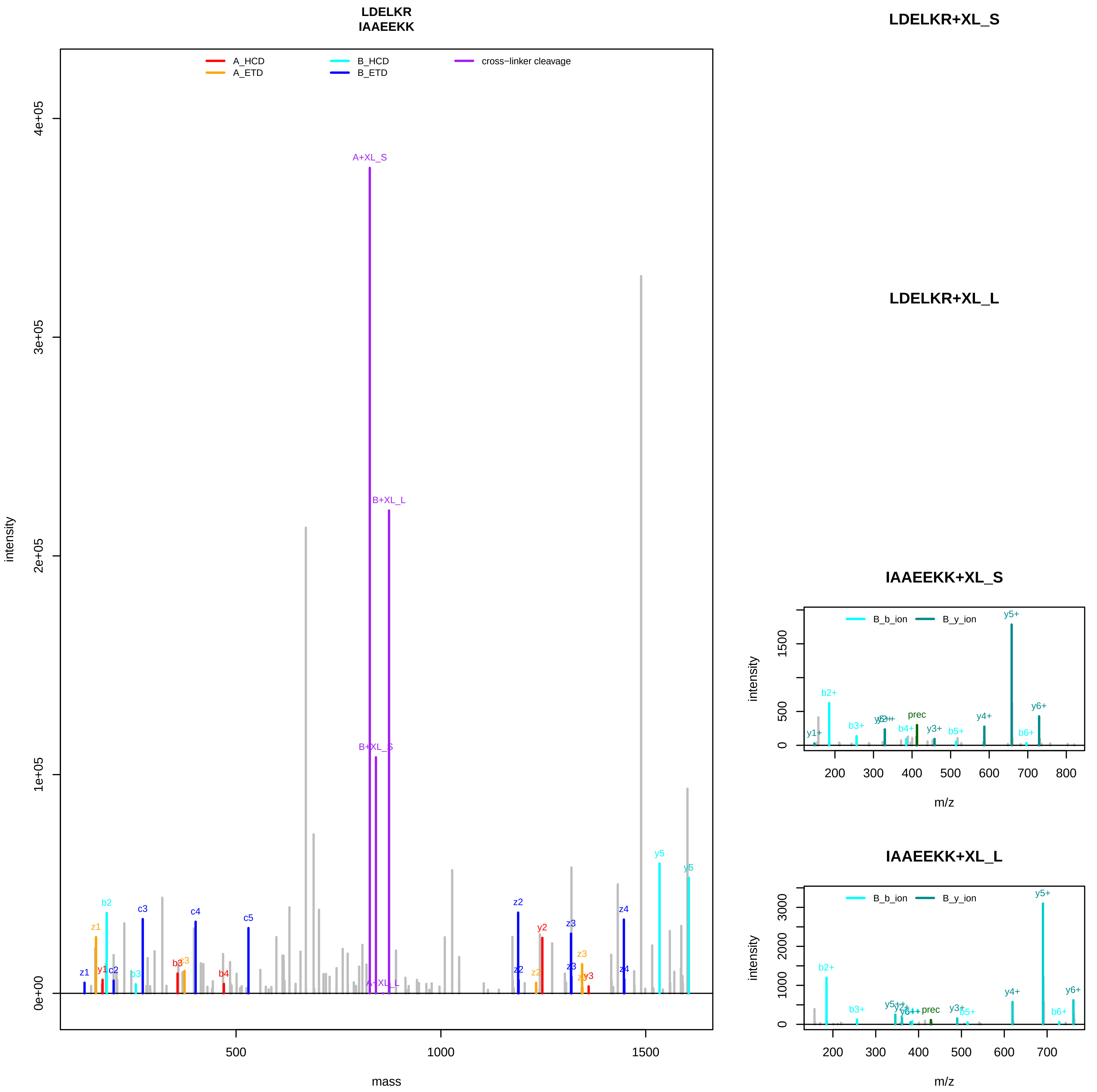
LDELKR
IAAEEKK
A_HCD
A_ETD
B_HCD
B_ETD
cross−linker cleavage
intensity
0e+00
1e+05
2e+05
3e+05
4e+05
mass
500
1000
1500
A+XL_S
B+XL_L
B+XL_S
A+XL_L
z1
y1
c2
b2
b3
c3
b3
z3
c4
b4
c5
z2
z2
z2
y2
z3
z3
z3
y3
z4
z4
y5
y5
LDELKR+XL_S
LDELKR+XL_L
IAAEEKK+XL_S
B_b_ion
B_y_ion
0
500
1500
m/z
200
300
400
500
600
700
800
b2+
b3+
b4+
b5+
b6+
y1+
y3+
y4+
y5+
y6+
prec
IAAEEKK+XL_L
0
1000
2000
3000
200
300
400
500
600
700
b2+
b3+
b5+
b6+
y3+
y4+
y5+
y6+
y6++
prec
